# Rapid detection of African swine fever virus using Cas12a-based portable paper diagnostics

**DOI:** 10.1038/s41421-020-0151-5

**Published:** 2020-04-07

**Authors:** Shuhan Lu, Fang Li, Qiubing Chen, Jing Wu, Junyi Duan, Xinlin Lei, Ying Zhang, Dongming Zhao, Zhigao Bu, Hao Yin

**Affiliations:** 1 Department of Pathology, Frontier Science Center for Immunology and Metabolism, Medical Research Institute, Zhongnan Hospital of Wuhan University, Wuhan University, Wuhan, China; 2 State Key Laboratory of Veterinary Biotechnology, National High Containment Laboratory for Animal Diseases Control and Prevention, Harbin Veterinary Research Institute, Chinese Academy of Agricultural Sciences, Harbin, China; 3 Medical Research Institute, Renmin Hospital of Wuhan University, Wuhan, China

**Keywords:** Biological techniques, Molecular biology, Biological techniques, Molecular biology

## Abstract

African swine fever virus (ASFV) is a dsDNA virus responsible for a severe, highly contagious, and lethal disease affecting both domestic and wild pigs. ASFV has brought enormous economic loss to a number of countries, and effective vaccine and therapy are still lacking. Therefore, a rapid, sensitive, and field-deployable detection of ASFV is important for disease surveillance and control. Herein, we developed a Cas12a-mediated portable paper assay to rapidly and precisely detect ASFV. We identified a robust set of crRNAs that recognized the highly conserved region of essential ASFV genes. The Cas12a-mediated detection assay showed low tolerance for mismatch mutations, and no cross-reactivity against other common swine pathogens. We further developed a paper-based assay to allow instrument-free detection of ASFV. Specifically, we applied gold nanoparticle–antibody conjugate to engineer homemade strips and combined it with Cas12a-mediated ASFV detection. This portable paper, instrument-free diagnostics, faithfully detected ASFV in swine samples, showing comparable sensitivity to the traditionally instrument-dependent qPCR method. Taking together, we developed a highly sensitive, instant, and economic Cas12a-mediated paper diagnostics of ASFV, with a great application potential for monitoring ASFV in the field.

## Introduction

African swine fever (ASF) is a highly contagious disease of swine that poses enormous economic losses due to its high mortality rate and rapid spread^1,2^. ASF has spread into a number of countries in Africa, Europe, and Asia during the past decade, with the possibility of further expansion^1,3^. The causative agent, ASF virus (ASFV), is a large double-stranded DNA virus belonging to the family *Asfarviridae*^2,4,5^. Due to the lack of vaccine and effective treatments against ASFV, disease control mainly relies on culling pigs^1,4^. More than 30 million domestic pigs were culled in the past 2 years, and the number continues to increase^6^. Thus, rapid diagnosis of the ASFV-affected animal is crucial to prevent its broad expansion, and to reduce economic losses^7,8^.

Existing ASFV detection methods recommended by World Organization for Animal Health (OIE) rely on virus isolation, antigen measurement by fluorescent antibody tests (FAT), or viral genome detection by polymerase chain reaction (PCR)^9,10^. Although virus isolation is the gold standard to diagnose ASFV, this time-consuming and complicated procedure is not applicable for real-time monitoring diseases^11^. Detection of ASFV antigen enables testing samples on a large scale, but it is not sensitive enough in detecting early-stage infection^8^. Both quantitative PCR (qPCR) and conventional PCR are fast and sensitive methods for detecting ASFV^9,11–13^. However, PCR methods require skilled operation and thermocyclers, making them less suitable for the field applications^14^. Isothermal amplification allows for easier operation and rapid amplification of DNA at a constant temperature independent of lab instruments^8,15–18^. However, nonspecific DNA amplification and high false-positive rates remain a big concern^15^. Therefore, a specific, sensitive, rapid, and equipment‐free assay is urgently demanded for monitoring ASFV in the field.

Clustered regularly interspaced short palindromic repeats (CRISPR)–CRISPR associated (Cas) systems recognize and cleave specific nucleic acid sequences (namely *cis*-cleavage)^19,20^. Some Cas proteins, including Cas12a, Cas12b, Cas13a, and Cas14, exhibit noncanonical *trans*-cleavage activity (alternative name “collateral effect”)^21–24^. Upon activation via recognizing a specific target sequence, these proteins exhibited the collateral effect by cleaving nontarget sequences^21–24^. Cas13a, an RNA-guided RNA endonuclease, gets activated via binding with sequence-specific RNA substrates. Activated Cas13a developed its nonspecific ribonuclease activity to degrade nearby single-stranded RNAs^23^. By combining the *trans*-cleavage activity of Cas13a with isothermal amplification and in vitro transcription of DNA to RNA, Cas13a‐based platform enables detection of nucleic acid at attomolar sensitivity^25^. When engineered with lateral flow for visual readout, it can generate a portable, rapid, and sensitive detection platform^26,27^. However, the in vitro transcription process is time-consuming, and the RNA probe used in the assay is unstable and expensive, limiting its broad applications. Cas12a, an RNA-guided DNA endonuclease, recognizes dsDNA, and upon activation, exhibits its nonspecific single stranded DNA (ssDNA) endonuclease activities^21,28^. Combining the collateral effect of Cas12a with isothermal amplification and a fluorescence readout has created a rapid and specific detection platform^21^. Cas12a-based detection assay is more straightforward than Cas13a, and its DNA probe is more stable and cost-effective. However, the fluorescence readout of Cas12a-based assay requires lab instruments. According to a recent report, the detection signal can be observed by naked eye under blue light^29^. This procedure is not suitable for detection in the field, as it requires a centrifuge, and its signal can be interfered in biological samples.

In this study, we developed to rapidly detect ASFV with high specificity and sensitivity by integrating Cas12a-based detection and gold nanoparticle-based lateral flow strip (named as Cas-gold). We compared it with the qPCR method using samples taken from ASFV-infected swine. The detection limit of two assays is comparable, and the test results of all samples are consistent. Our data indicate that Cas-gold is a useful method to monitor ASFV in the field.

## Results

### Select robust crRNA against the conservative regions of ASFV

**Fig. 1:**
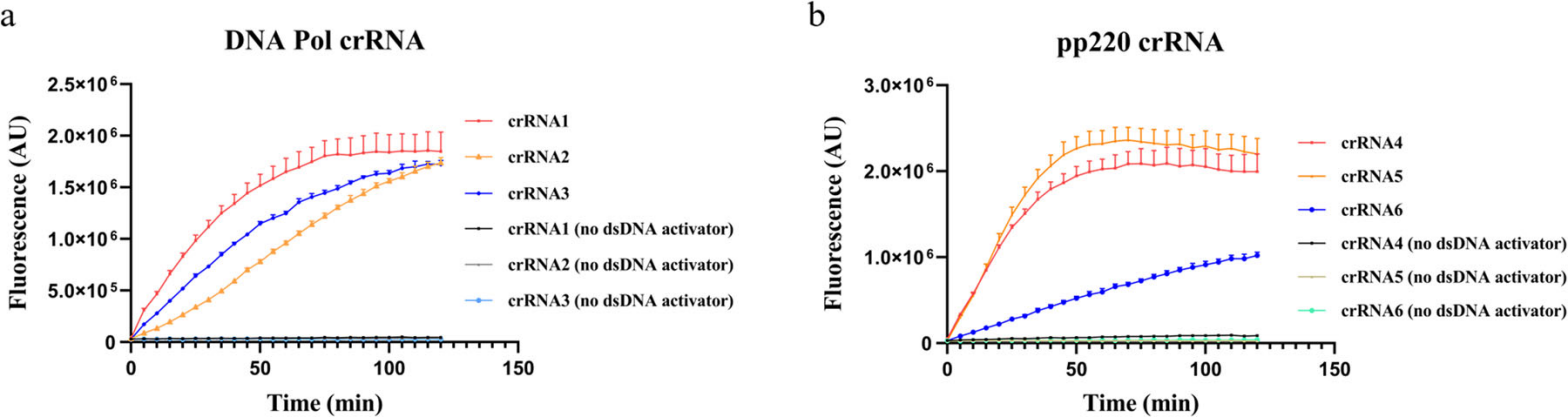
crRNAs targeting DNA polymerase and pp220 gene of ASFV. Fluorescence detection using crRNAs targeting the highly conserved region of **a** DNA polymerase gene, and **b** pp220 gene and ssDNA-FQ reporter. Error bars represent mean ± SD, *n* = 3.

The crRNA design has played an essential role in the sensitivity of Cas12a-based viral detection^21–24^. It is critical to establish a single crRNA strategy that monitors most ASFV subtypes via the CRISPR-based detection^9,11–13^. To achieve this goal, we designed six crRNAs by either targeting the conserved regions of polyprotein pp220 (briefly as pp220) or DNA polymerase gene (DNA Pol), both of which are essential to ASFV life cycle^2,5^ (Supplementary Table S1). The ability to specifically cleave pp220 or DNA Pol double-stranded DNA (dsDNA) sequences by Cas12a–crRNA complex was demonstrated by in vitro cleavage activity assay (Supplementary Fig. S1). To confirm the *trans*-effect of Cas12a after target recognition, a ssDNA, FAM-Quencher reporter, was designed and synthesized (FAM-TTATT-Quencher, as ssDNA-FQ reporter). Upon ssDNA cleavage by Cas12a, the FAM signal is released and measured. The assay showed little background when the ssDNA-FQ reporter is intact (Supplementary Fig. S2a, b). When DNA Pol or pp220 dsDNA sequences were presented, the crRNA1-targeted DNA Pol and crRNA5-targeted pp220 outperformed other crRNAs in terms of fluorescence intensity and activity kinetics (Fig. 1a, b). Therefore, crRNA1 targeting DNA Pol and crRNA5 targeting pp220 were selected for further experiments.

### The specificity of Cas12a-mediated detection platform

**Fig. 2:**
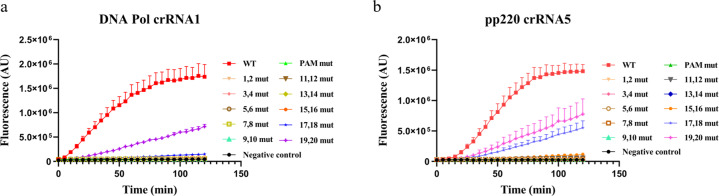
The mismatch mutation tolerance of Cas12a-based detection system. Fluorescence detection using crRNA-targeting perfect match (WT) or mutated dsDNA. The crRNA1 (**a**) and crRNA5 (**b**) targeting sequences were mutated, respectively. **a** The PAM (TTTG→AGCG) and PAM-proximal 1–20-nt sequences were mutated, respectively. **b** The PAM (TTTA→AGCA) and PAM-proximal 1–20-nt sequences were mutated, respectively. Error bars represent mean ± SD, *n* = 3.

To determine the specificity of Cas12a-based fluorescence detection, we introduced a series of two-nucleotide mutations to the target DNA. Either the PAM or the DNA sequences complementary to the guide sequences of crRNA1 and crRNA5, respectively, were mutated. Compared with no dsDNA substrate control, little fluorescence signal was detected in mutated dsDNA when PAM or PAM-proximal sequences were changed (Fig. 2a, b). Only mutations in the PAM-distal 19–20 nt for crRNA1 and crRNA5, and in the PAM-distal 17–18 group for crRNA5, showed substantial fluorescence signal (Fig. 2a, b), indicating the high specificity of Cas12a-based detection for ASFV sequences. To further investigate whether Cas12a-based fluorescence detection could tolerate point mutation, a series of 1-nt mutations were introduced into the target DNA. Consistent with the 2-nt mutation data, we observed a much reduced fluorescence signal in the point mutation of 17 nucleotides next to PAM, and the point mutation of the PAM-distal 18–20 still showed substantial fluorescence signal (Supplementary Fig. S3a, b). However, we observed more than background fluorescence signals in several point-mutated sequences, suggesting that detection systems based on Cas12a may partially tolerate point mutation at certain positions. These data are consistent with a previous study, indicating that Cas12b-based nucleic acid detection platform partially tolerated point mutation^30^. We also detected the fluorescence signal of several 1-nt deletion or insertion sequences, and these sequences showed little fluorescence signal (Supplementary Fig. S3c).

**Fig. 3:**
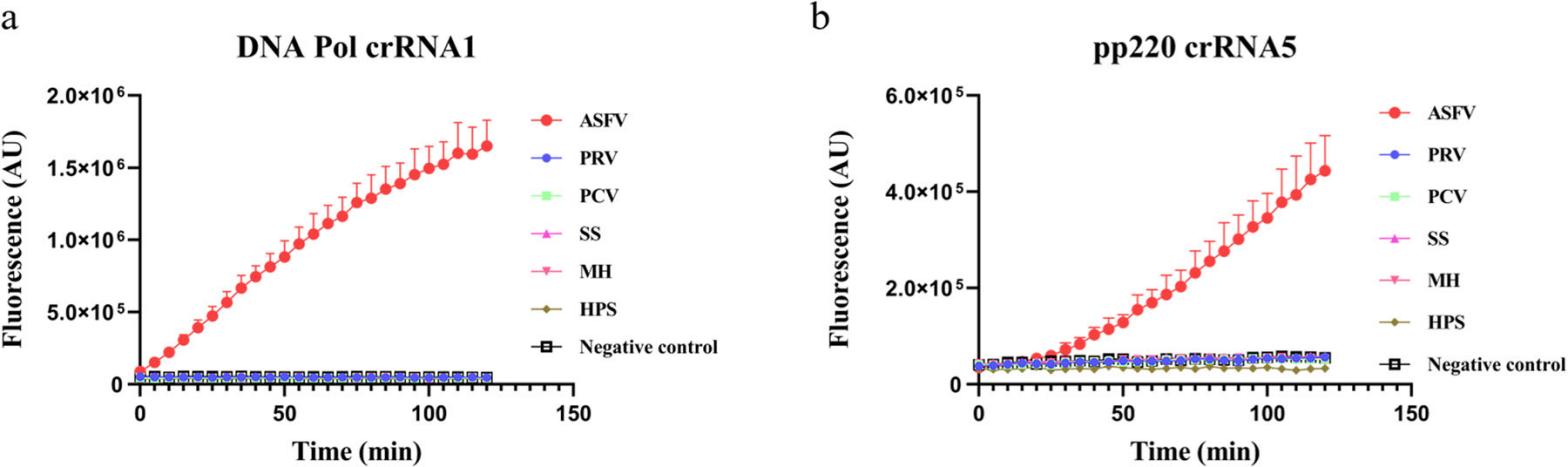
Specificity of Cas12a-based detection system. Fluorescence detection using **a** crRNA1 and **b** crRNA5 to detect ASFV and five other swine DNA pathogens (PRV, PCV, SS, MH, and HPS). Error bars represent mean ± SD, *n* = 3.

To further determine the specificity of Cas12a-based detection of ASFV against other viruses, several common swine pathogens, including Pseudorabies virus (PRV), Porcine circovirus (PCV), Streptococcus suis (SS), Mycoplasma hyorhinis (MH), and Haemophilus parasuis (HPS), were propagated, and their genomic DNA was extracted for detection. Both DNA pol crRNA1 and pp220 crRNA5 showed a high and specific fluorescence signal against ASFV, whereas other pathogen viruses triggered few signals (Fig. 3a, b). Since it is possible that veterinary samples are contaminated by human viruses, we put the guide sequences of crRNA1 and crRNA5 in nucleotide BLAST, and analyzed the top comparison results. All viruses other than ASFV from the analysis showed less than 60% sequence similarity, suggesting low target sequence similarity of swine and human viruses (Supplementary Table S2). Together, these data demonstrated that the Cas12a/crRNA-based detection platform is specific for ASFV.

### Cas12a-mediated fluorescence detection of veterinary samples

**Fig. 4:**
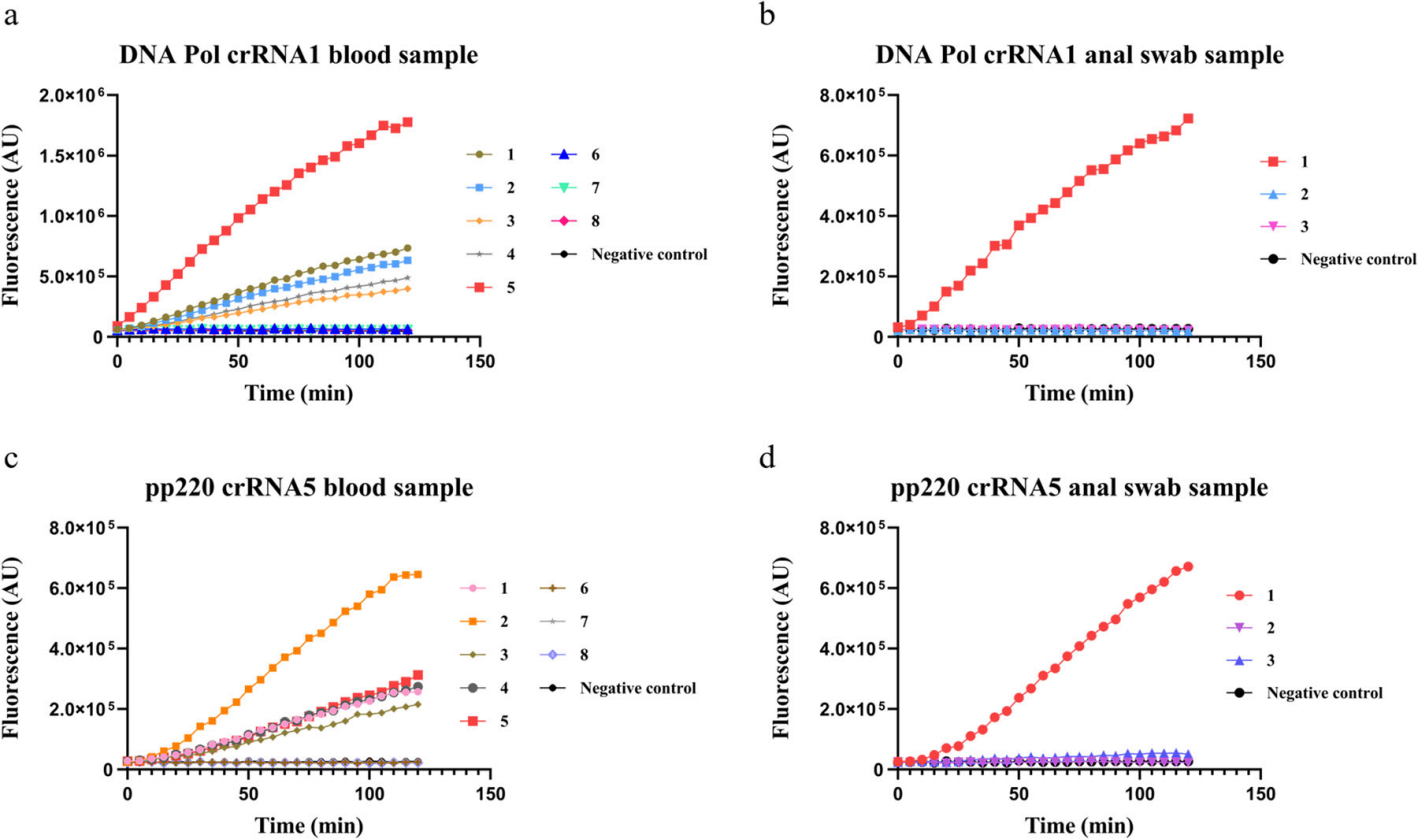
Cas12a-based fluorescence detection of veterinary samples. Genomic DNA was extracted from swine blood or anal swab. Blood samples 1–5 or 6–8 were determined by qPCR as positive or negative of ASFV, respectively. Anal swab samples 1 or 2–3 were determined by qPCR as positive or negative of ASFV, respectively. PCR amplification of DNA Pol and pp220 genes was performed. The genomic DNA from blood (**a**, **c**) or anal swab (**b**, **d**) was used. Fluorescence were measured for 120 min after incubation with Cas12a, ssDNA reporter, and crRNA1 (**a**, **b**) or crRNA5 (**c**, **d**).

To explore the veterinary potential of the detection platform, we extracted DNA from blood and anal swabs of ASFV-infected pigs. The crRNA recognition sequences were enriched via PCR, and were then incubated with Cas12a/crRNA complex in the presence of the ssDNA-FQ reporter. The presence of ASFV was first determined by qPCR, which is widely adopted to detect ASFV in the laboratory. As shown in Fig. 4a–d, all positive samples, including blood samples (blood samples 1–5) and swab samples (swab sample 1), showed a strong fluorescence signal by using crRNA1 or crRNA5; all negative samples (blood samples 6–8 and swab samples 2 and 3) showed no difference compared with negative control (no dsDNA). The high detection accuracy suggests that Cas12a-based detection of ASFV is viable.

### RPA and lateral flow detection

**Fig. 5:**
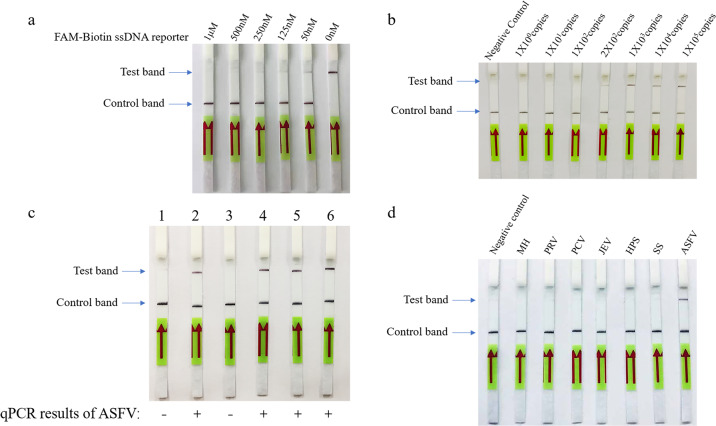
Rapid and visual detection of ASFV. **a** Determine the optimum amount of FAM-Biotin ssDNA reporter. **b** Determine the detection limit of ASFV genome copy numbers by using the combination of RPA, Cas12a, and lateral flow. **c** Detect ASFV in swine samples. Samples 1, 3, or 2, 4–6 were confirmed by qPCR as negative or positive of ASFV, respectively. The samples 1–6 were 10, 15, 21, 23, 24, and 26 listed in Table 1. The viral copy numbers of the samples 2, 4, 5, and 6 determined by qPCR were 6.84E + 02, 2.97E + 02, 6.90E + 03, and 1.96E + 03 copies/µl, respectively. For the visual detection assay, crRNA1 was applied. **d** Determine the specificity of Cas12a-based lateral flow by examining ASFV and other porcine pathogens (MH, PRV, PCV, JEV, HPS, and SS).

One biggest disadvantage of fluorescence-based detection is the requirement of special lab equipment, which is not available to farmers. Given the rapid spread and highly contagious characteristics of ASFV, it is crucial to develop a real-time detection method that can monitor ASFV in the field, and be operated by farmers. We combined recombinase polymerase amplification (RPA) technique and the lateral flow detection technique to replace the instrument-dependent PCR amplification technology and fluorescence detection method. RPA allows rapid amplification independent of the instrument, whereas the lateral flow detection allows visual readout of test results. We optimized the primers of RPA to make it robust (Supplementary Table S3). The lateral flow detection technique was described as the following. Au nanoparticles conjugated with an anti-FITC antibody were on the binding pad. The streptavidin and IgG were fixed on the NC membrane, as a control line to specifically bind biotin, and test line to specifically bind an anti-FITC antibody, respectively. The FAM-Biotin ssDNA reporter specifically bound to Au nanoparticles to form a complex because FAM can be recognized by the anti-FITC antibody on the Au nanoparticles. When ssDNA was not degraded by Cas12a, this complex bound to streptavidin at the control line. On the contrary, when ssDNA was degraded to cause Biotin release from the complex, the complex could pass through the control line, and bound to the IgG antibody on the test line. When we applied the concentration of reporters used in fluorescence assay for lateral flow detection, an obvious false-positive band was shown in blank control (Fig. 5a). We believed that a portion of nanoparticles did not bind to the FAM-Biotin ssDNA reporter; thus, they kept moving forward until they reached the test band to make the potential false-positive result. Various concentrations of FAM-Biotin ssDNA reporter were added. A higher concentration of FAM-Biotin ssDNA reporter gradually diminished the false-positive band (Fig. 5a). Thus, we chose 1 μM as the concentration of reporter for the next set of experiments. When we used the concentration of LbCas12a/crRNA complex used in fluorescence assay for lateral flow detection, the test band appeared in positive samples (Supplementary Fig. S4). However, the band was fuzzy despite the extended reaction time. When we increased the concentration of LbCas12a/crRNA complex, the test band was striking in positive samples, but it was not shown in negative samples (Supplementary Fig. S4). To examine the optimal reaction time and temperature of Cas12a-based lateral flow, we compared reaction times of 1, 2, 5, and 10 min, and reaction temperature of 4 °C, room temperature and 37 °C. We identified that 1- or 2-min reaction time was sufficient to observe a clear test band, and the reaction at room temperature showed a clearer band than at 4 and 37 °C (Supplementary Fig. S5).

To determine the detection limit of the assay, ASFV genome ranging from 1 to 10^5^ copies was tested. A clear positive band started to present at 2 × 10^2^ copies, suggesting that the detection limit is as low as 200 copies of viral genome (Fig. 5b). This is very similar to the detection limit of qPCR-based approach (Supplementary Fig. S6), showing that an engineered instrument-free CRISPR-based strip assay has comparable sensitivity to the instrument-dependent method. To determine whether using multiple crRNAs could increase the sensitivity, DNA Pol crRNA1 and pp220 crRNA5 were used simultaneously in one reaction. The detection limit remained as 2 × 10^2^ copies (Supplementary Fig. S7). Thus, we kept using crRNA1 for the following experiments. To determine the accuracy of the assay, 30 veterinary samples collected from blood, oral, or anal swabs were tested. Consistent with qPCR results, all negative samples showed the control band only in the strip, and all positive samples showed both control and test bands (Fig. 5c and Table 1). To examine the specificity of this assay, swine pathogens, such as PRV, PCV, SS, MH, HPS, and Japanese encephalitis virus (JEV), were tested by the strips. All these swine pathogens were shown negative, while only ASFV was positive (Fig. 5d).

**Table 1:** Detection of ASFV in swine samples by Cas12a-based strip and qPCR.

Sample	Source	Copies/µl by qPCR	Cas12a-based strip
1	Blood	8.62E + 06	Positive
2	Blood	9.99E + 05	Positive
3	Blood	1.16E + 06	Positive
4	Blood	5.87E + 06	Positive
5	Blood	5.55E + 06	Positive
6	Blood	2.43E + 07	Positive
7	Blood	3.00E + 06	Positive
8	Blood	1.29E + 06	Positive
9	Blood	–	Negative
10	Blood	–	Negative
11	Blood	–	Negative
12	Oral swab	6.19E + 02	Positive
13	Oral swab	3.64E + 02	Positive
14	Oral swab	3.32E + 02	Positive
15	Oral swab	6.84E + 02	Positive
16	Oral swab	2.98E + 03	Positive
17	Oral swab	1.01E + 03	Positive
18	Oral swab	4.61E + 03	Positive
19	Oral swab	3.04E + 03	Positive
20	Oral swab	1.66E + 03	Positive
21	Oral swab	–	Negative
22	Anal swab	1.34E + 03	Positive
23	Anal swab	2.97E + 02	Positive
24	Anal swab	6.90E + 03	Positive
25	Anal swab	1.47E + 03	Positive
26	Anal swab	1.96E + 03	Positive
27	Anal swab	1.10E + 03	Positive
28	Anal swab	1.08E + 03	Positive
29	Anal swab	–	Negative
30	Anal swab	–	Negative

### Engineer homemade test strips

**Fig. 6:**
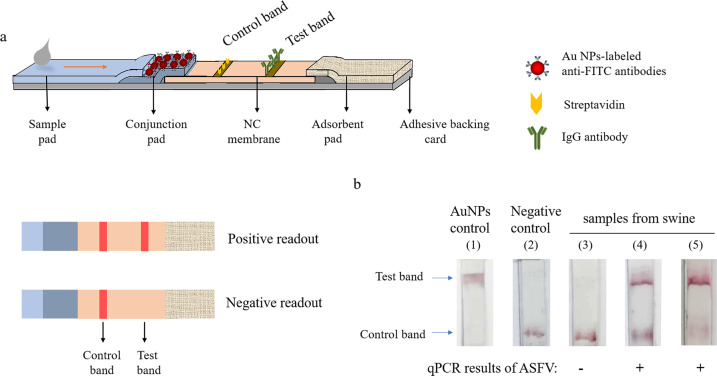
Engineer homemade immune-chromatographic test strips for Cas12a-based ASFV detection. **a** Schematic diagram of immunochromatographic test strips. **b** Strip 1: confirm the penetrability of gold nanoparticles coupled with anti-FITC antibodies in strips. Strip 2: negative control (no DNA sample in RPA reaction buffer). Strips 3–5: lateral flow detection of samples from swine. Determined by qPCR, sample 3 was negative, and the viral copies of samples 4 and 5 were 6.84E + 02 and 1.29E + 06 copies/µl, respectively.

The high cost of a commercial strip makes the detection method less applicable to the general public. To solve this, we engineered a homemade immunochromatographic strip using gold nanoparticles (Au NPs) (Fig. 6a). When tested with swine samples, the homemade strips faithfully recapitulated the results similar to the commercial strip, whereas the cost is less than 1% of the commercial strip (named as Cas-gold detection) (Fig. 6b).

## Discussion

Since 2018, the rapid outbreak of ASF in China and a number of other countries has resulted in tremendous economic losses^2,6^. Due to the lack of effective vaccine and treatment, the development of a real-time ASFV detection method is critical to limit the disease expansion at an early stage^7,8^. CRISPR–Cas systems are revolutionary tools allowing for precise genome engineering, transcription regulation, and many other applications^31–33^. The *trans*-activity of several class 2 Cas endonucleases allows sensitive and robust detection of nucleic acids^21–24^. Among them, Cas12a recognizes specific dsDNA sequences, and then nonspecifically cleaves ssDNA, making it particularly suitable for detecting dsDNA viruses^21^. In combination with isothermal amplification, we found that Cas12a was able to detect ASFV with high sensitivity and precision. It is possible that other members of Cas12 family can also be used for ASFV detection. Cas12b-mediated DNA detection was recently developed, and it showed higher sensitivity than Cas12a on several examined sequences^34^. It may be useful to evaluate the potential of Cas12b for detecting ASFV in the future.

Our results indicate that the Cas12a-based detection system has low tolerance for base-pair mutations (Fig. 2). The PAM and PAM-proximal sequences are usually more important than the PAM-distal for target recognition of dsDNA^35^. In consistent, our data showed that the PAM sequence and PAM-proximal 16–18-nt sequences (taking together 20–22 nt) did not tolerate 2-nt mismatch mutations (Fig. 2). The stringent system may assure that it does not cross-react with other swine pathogens. Indeed, when tested with five other common swine pathogens, none of them showed any fluorescence signal (Fig. 3). It is useful to understand the mutation frequencies of the DNA Pol and pp220 genes, particularly their conservative regions for precise detection of ASFV. However, this information was rarely reported, and further investigations are demanding^36,37^.

We noticed that a number of RPA primers for pp220 and DNA Pol amplified the target sequences when substrates were plasmids. However, most of these primers could not amplify the target sequences from swine samples (data not shown). Only a few RPA primer pairs consistently work for all ASFV-positive swine samples (Supplementary Table S3).

Cas12a has been engineered to detect pathogens in clinical samples^21^. However, a fluorescence reporter was used in the study, which restricted its application in the laboratories^21^. A recent study developed a blue light-based readout^29^. Though this readout does not rely on laboratory equipment, it is less applicable in the field. We aimed to develop a strip-based, high-sensitivity assay to detect ASFV independent of lab instruments. We noticed that the conditions of Cas12a fluorescent assay cannot be directly applied for strip detection (Fig. 5). After a thorough optimization of various parameters, we successfully generated a robust strip-based assay to fulfil the detection potential of Cas12a (Fig. 5). The sensitivity of this assay is as low as 200 copies/sample, which is comparable as qPCR assay executed in laboratories (Fig. 5; Supplementary Fig. S6). The low detection limit of the assay allows the diagnosis of ASF at the early stage of infection^38^. It is more sensitive and accurate than other field-applicable assays, which are usually based on isothermal amplification alone^11^. The strip can be kept in dry environment at 4 °C for at least 3 months, as previously reported, making it suitable for commercial use^39–41^.

**Fig. 7:**
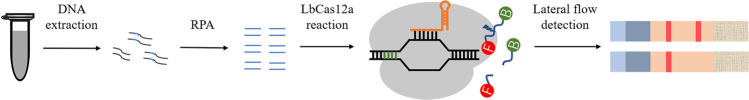
Schematic workflow of Cas12a-based detection for ASFV (namely Cas-gold). ASFV genome was extracted from the swine blood or swab samples and amplified by RPA. It was incubated with crRNA/Cas12a complex and FAM-Biotin ssDNA reporter to perform *trans*-cleavage. The detection results could be read in test strips.

Despite the robust results, the high cost of commercial strips limits their broad application for ASFV detection. To overcome this limitation, we successfully engineered a homemade strip using gold nanoparticle–antibody conjugate (named as Cas-gold). For the homemade version of strips, all parts were completed by hand. Because the detection line was made with multiple microspots, the strips had more smear bands than commercial ones. In the commercial production, this step can be completed by professional equipment to avoid this issue. Nevertheless, we showed that the homemade strip exhibited comparable results to the commercial strip (Fig. 6). Homemade strip provides a robust, easy, and cost-effective alternative. In summary, our study developed a field-deployable, cost-effective, highly sensitive, and accurate Cas12a-based strip method to faithfully detect ASFV (Fig. 7).

During the period of manuscript revision, the outbreak of COVID-19 coronavirus began in Wuhan city, leading to more than 3,000 deaths and 80,000 hospitalizations so far. Sustained person-to-person spread of COVID-19 was reported, and it may cause severe pneumonia and other complications in people^42^. The Cas12a-based strip may be useful to rapidly detect COVID-19, particularly in regions that lack resources.

## Materials and methods

### Facility and ethics statements

Animal experiments and cell culture with live ASF viruses were performed in the enhanced biosafety level 3 (P3+) and level 4 (P4) facilities, both of which are approved by the Ministry of Agriculture and Rural Affairs. These facilities are in the Harbin Veterinary Research Institute (HVRI), supervised by the Chinese Academy of Agricultural Sciences (CAAS). These studies were accomplished in strict accordance with the recommendations in the Guide for the Care and Use of Laboratory Animals of the Ministry of Science and Technology of the People’s Republic of China, and were approved by the Animal Ethics Committee of HVRI and the Animal Ethics Committee of Heilongjiang Province, China.

### ASFV growth and animal experiments

Primary porcine alveolar macrophages were infected at a multiplicity of infection (MOI) of 0.1 with ASFV, which was isolated from field samples as described previously^43,44^. Cell supernatants were collected on day 3 after infection. Seven-week-old SPF Large White and Landrace-crossed pigs were acquired from the Laboratory Animal Center of HVRI. Pigs were intramuscularly inoculated with ASFV at a dose of 10^2.5^ HAD_50_, respectively. Blood, oral or anal swabs were collected daily for virus detection.

### Reagent for molecular assays

All primers were ordered in Sangon Biotech (Shanghai, China), and the detailed sequences were listed in Supplementary Table S3. FAM-TTATT-Quencher used in fluorescent reporter assay was synthesized by Integrated DNA Technologies (IDT); FAM-TTATT-Biotin used in lateral flow strip test and probe in qPCR assay were ordered in TaKaRa Bio Inc. (Dalian, China). T7 RNA polymerase and NEBuffer 2.1 were purchased from New England Biolabs (MA, USA). The TwistAmp® Basic kit and Milenia HybriDetect 1 were purchased from TwistDx (Cambridge, UK). 2× AceQ qPCR Probe Master Mix and Phanta High-fidelity DNA polymerase were purchased from Vazyme (Nanjing, China).

### LbCas12a expression and purification

The DNA fragment encoding LbCas12a was cloned into the pET30c vector containing the C-terminal 6× His tag to construct an expression plasmid, and then transformed into *E. coli* BL21 (DE3). Cells were transferred to 1 L of 2× YT medium (10 g of tryptone, 10 g of yeast extract, and 5 g of NaCl). Two hours later, the temperature was adjusted from 37 to 21 °C, and 0.5 mM IPTG was added. Sixteen hours later, LbCas12a was purified from the cell lysate via Ni-NTA column (HisTrap HP, GE) and the size-exclusion chromatography.

### crRNA preparation and in vitro cleavage assay

The sequence of DNA Pol and pp220 gene from 30 different ASFV genomes were downloaded from NCBI database, and aligned to determine the highly conserved regions. The crRNAs were designed against the conserved regions using the online software (CCTOP, https://crispr.cos.uni-heidelberg.de/). The top crRNAs for the target regions were selected to perform in vitro cleavage assay. DNA sequences containing T7 promotor (Supplementary Table S3) were synthesized and annealed to form dsDNA templates for in vitro transcription. The crRNAs were synthesized by T7 RNA polymerase followed by DNase I digestion. For in vitro cleavage assay, the crRNA–Cas12a complex was formed by incubating 500 ng of crRNA and 500 ng of LbCas12a for 10 min at 25 °C. DNA substrate (150 ng) was added and co-incubated at 37 °C for 30 min.

### dsDNA activator preparation

DNA Pol and pp220 fragments were cloned into PUC57 vector via Gibson assembly. The plasmids PUC57-DNA Pol and PUC57-pp220 were used as dsDNA activators to select robust crRNAs. The wild-type, PAM-mutated, and target sequence-mutated oligos were synthesized (Supplementary Table S3). The non-targeted strand to target strand was annealed at 50:1 molar ratio.

### Fluorescence-quencher (FQ)-labelled reporter assay

The Cas12a-mediated FQ-labelled reporter assay was performed using 6 ng of crRNA, 50 ng of Cas12a, 50 nM quenched fluorescent ssDNA reporter, and 20 nM plasmid activators in a 30-μL reaction volume. The reaction was performed at 37 °C for 120 min on a fluorescence reader, and the fluorescence values were measured every 5 min (*λ*_ex_: 485 nm; *λ*_em_: 535 nm).

### ASFV genome DNA sample preparation and fluorescence detection

Viral DNA was extracted via GenElute™ Mammalian Genomic DNA Miniprep Kits (Sigma Aldrich, USA) from blood, swabs and cell supernatants. ASFV genomic DNA extracted from different samples was quantified by using qPCR assay, according to the OIE-recommended procedure described^45^. Titers of virus were determined by detecting viral copy numbers of p72 gene via qPCR. To perform fluorescence detection, samples were first amplified using PCR, and then incubated with LbCas12a. Fluorescence detection was performed as described above, and 8 µl of PCR product was used in a 30-μL reaction volume.

### RPA reaction and lateral flow strip detection

RPA primers were designed using the online software (Primer-blast). These forward and reverse primers formed a number of primer pairs, and the RPA products should be 100–200 bp. The capability and stability of amplifications were determined using veterinary samples with low and high viral copies (1.0E + 03 and 1.0E + 05), and the best-performed primer pair was selected. Reaction temperatures of 37, 39, and 42 °C were examined, and various concentrations of magnesium acetate were tested. The RPA reaction, more stable, was performed at 39 °C, and by using 28 mM magnesium acetate. The RPA reaction was performed according to the manufacturer’s instruction. The RPA mixture was mixed with 150 ng of LbCas12a, 18 ng of crRNA, 1 μM ssDNA (FAM-TTATT-Biotin) reporter, and 2 μL of NEBuffer 2.1. After 90 min of incubation, 160 μL of HybriDetect assay buffer was added, and the strips were inserted.

### Fabrication of immunochromatographic test strips

The gold nanoparticles (Au NPs) were prepared as previously described^46^. Briefly, sodium citrate solution (1%, w/v) was added into the boiling HAuCl_4_ solution (0.01%, w/v). After continuous stirring for 30 min, the reaction system was cooled to room temperature to obtain a stable colloidal gold suspension. Subsequently, the size of Au NPs was detected by dynamic light scattering. To further prepare the Au NP–antibody conjugate, NaOH solution was used to adjust the pH of Au NP suspension, and the anti-FITC antibody was added. After stirring for 1 hour at 37 °C, the bovine serum albumin (BSA) solution (0.1%, w/v) was added, and the Au NP–antibody conjugates were collected by centrifugation. The immunologic test strips were composed of a sample pad, a conjugate pad, an antibody pad, a NC membrane, and an adaptive backing card^46^. Briefly, the conjugate pad was dripped with appropriate amounts of Au NP–antibody conjugate suspension, and then dried for 1 hour at 37 °C. The streptavidin and nonspecific capture antibody (IgG) were dripped into the NC membrane to form control and test bands. The NC membrane was dried for 1 hour at 37 °C, and then was immersed in BSA solution (1%, w/v) to passivate the unbound sites, and dried for 1 hour at 37 °C. The sample pad, reconcile pad, NC membrane, and absolute pad were assembled on the adaptive backing card to form complete immunochromographic test strips.

## Supplementary information

Supplementary Table S2

Supplementary Figures

Supplementary Table S1

Supplementary Table S3

## Supplementary information

**Supplementary Information** accompanies the paper at (10.1038/s41421-020-0151-5).
