# Supplementary material for: Rapid detection of African swine fever virus using Cas12a-based portable paper diagnostics
Source: Cell Discov. 2020 Apr 7;6:18. doi: 10.1038/s41421-020-0151-5 (PMC7136273; doi:10.1038/s41421-020-0151-5)
Supplement: Supplementary file 2 — Supplementary Figures [file 41421_2020_151_MOESM2_ESM.pdf]

Supplementary Fig. S1

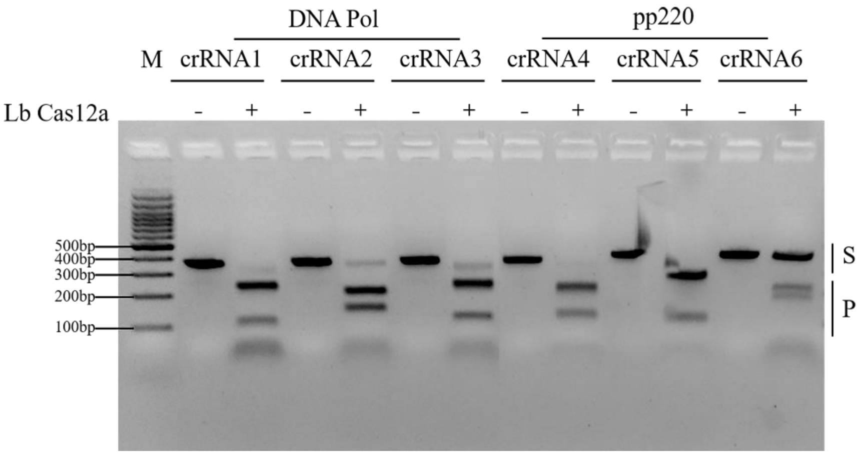

Supplementary Fig. S1. The crRNAs targeting the conservative regions of DNA Pol and pp220 were incubated with LbCas12a and dsDNA substrate to determine the cis-cleavage of dsDNA targets. The substrate (S) and cleavage products (P) were shown in agarose gel.

Supplementary Fig. S2

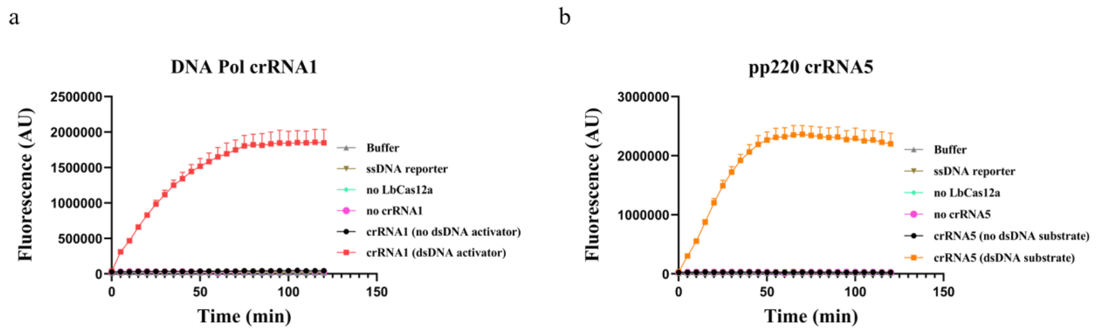

Supplementary Fig. S2. Fluorescence detection using (a) crRNA1 and (b) crRNA5.

Supplementary Fig. S3

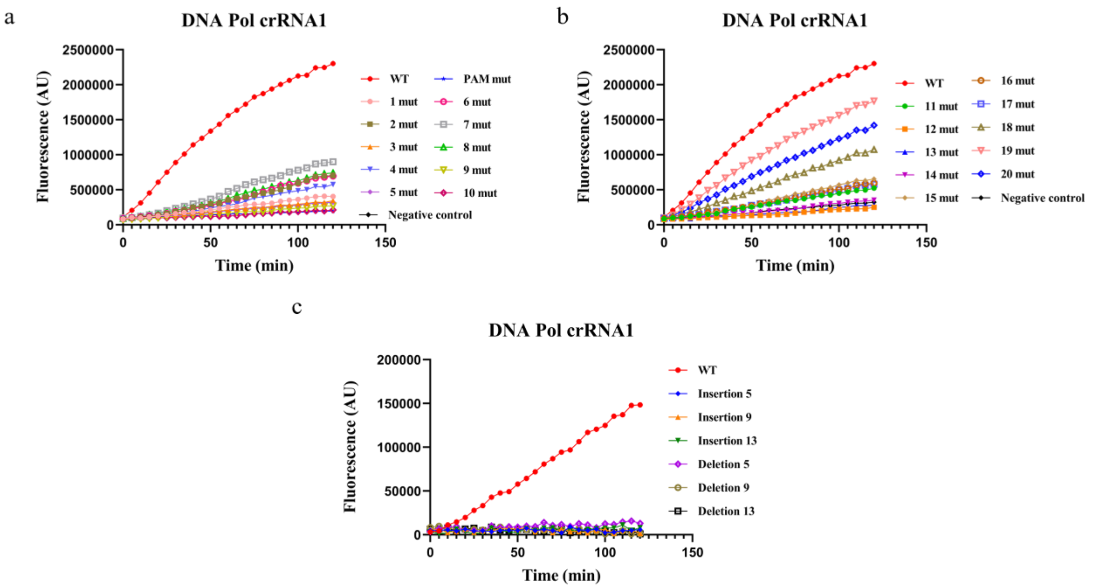

Supplementary Fig. S3. Single base mutation, insertion and deletion were introduced to the crRNA1 targeting sequences and fluorescence detection was performed. (a) The PAM-proximal 1-10 nt and (b) 11-20nt was mutated 1 base pair. (c) The 1-nt insertion and deletion in PAM-proximal 5, 9 and 13 nt were introduced.

Supplementary Fig. S4

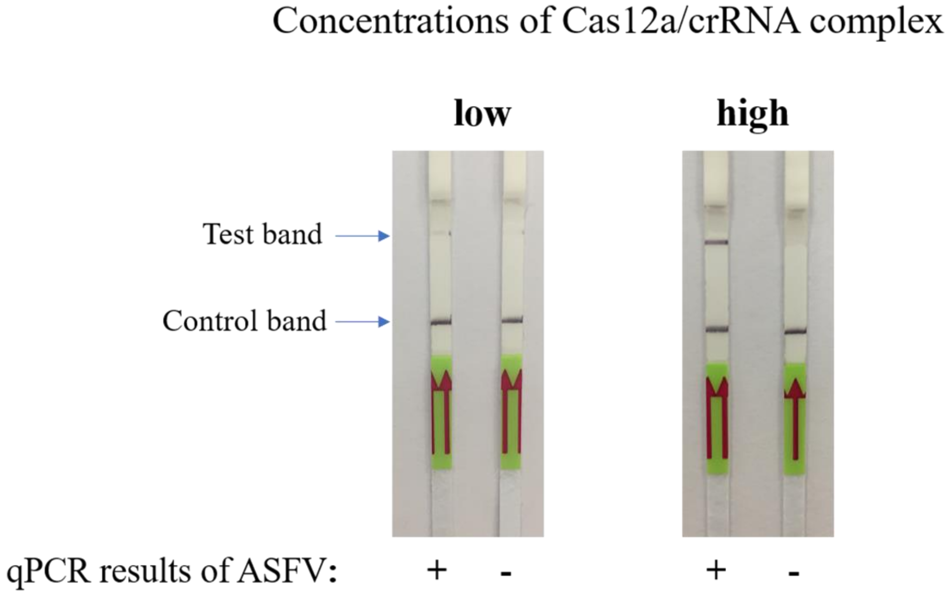

Supplementary Fig. S4. Determine the concentrations of Cas12a/crRNA complex for lateral flow detection. The concentrations of Cas12a/crRNA complex are 6 ng crRNA / 50 ng LbCas12a in “low” groups and 18 ng crRNA / 150 ng LbCas12a in “high”.

Supplementary Fig. S5

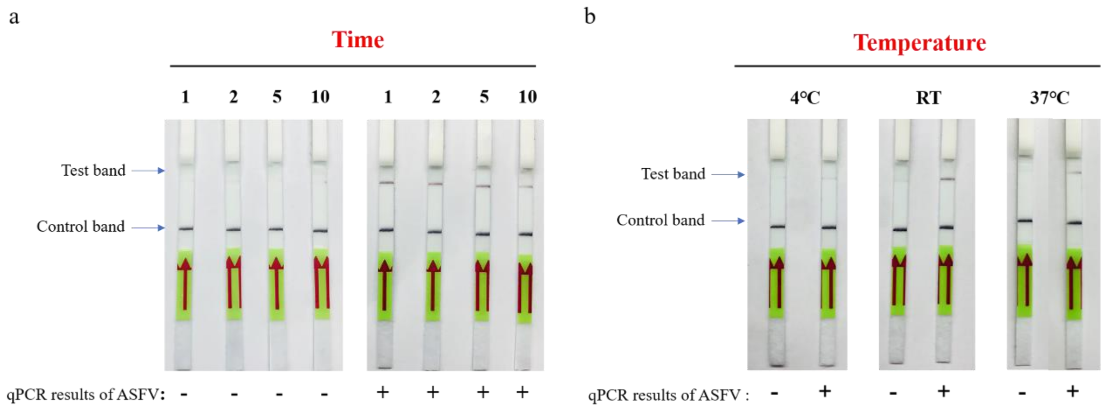

Supplementary Fig. S5. Examine the optimal reaction condition of Cas12a-based lateral flow, including (a) 1, 2, 5 and 10 minutes of reaction time and (b) 4℃, room temperature (RT) and 37℃.

Supplementary Fig. S6

a

C(copies/μL)	Cq
1.00E+06	21.1
1.00E+05	25.1
1.00E+04	29.3
1.00E+03	33.1
1.00E+02	37.1
5.00E+01	NA

b

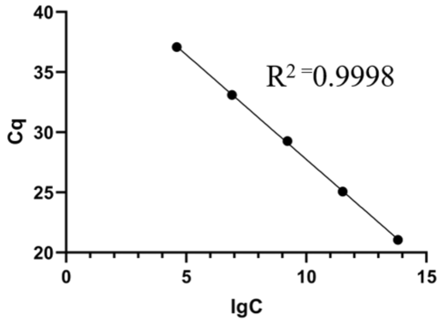

Supplementary Fig. S6. Determine the detection limit of qPCR assay. Copy numbers and Cq values were listed in (a) and standard curve was shown in (b).

Supplementary Fig. S7

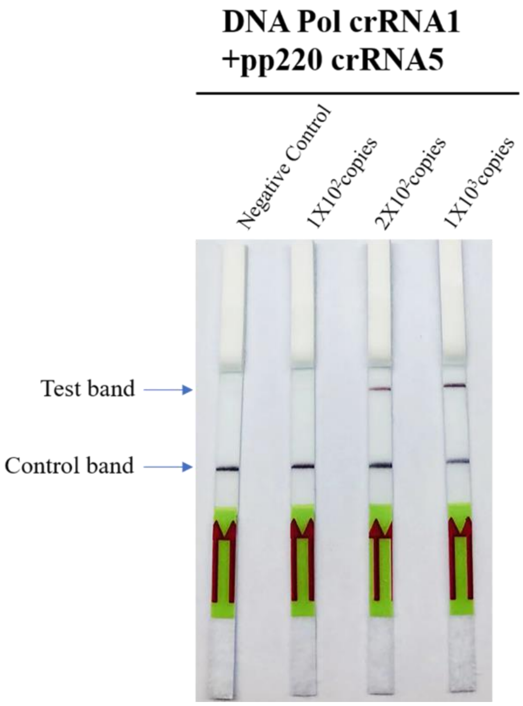

Supplementary Fig. S7. The crRNA1 and crRNA5 were combined in one reaction, and the detection limit of Cas12a-based lateral flow were determined.
