# Supplementary material for: Rapid detection of African swine fever virus using Cas12a-based portable paper diagnostics
Source: Cell Discov. 2020 Apr 7;6:18. doi: 10.1038/s41421-020-0151-5 (PMC7136273; doi:10.1038/s41421-020-0151-5)
Supplement: Supplementary file 3 — Supplementary Table S1 [file 41421_2020_151_MOESM3_ESM.pdf]

Supplementary Table S1. Conservative regions of target genes (DNA polymerase and pp220) and crRNA sequences. Partial sequences of DNA polymerase and pp220 gene are listed. Conservative regions are labelled in brackets and bold, and DNA sequences targeted by Cas12a/crRNAs are highlighted. The sequences of crRNAs are listed.

DNA Pol

…TATCCATGAAGTACGATATGGAGCCTACACACTTTTCATGTATGGTTCCCTCGAAAAC**[GGTTACAAAGCAGAAGTAAGGATTGAAAA**]CATCCCAGTTTTCTTTGACGTACAGATT…TGGGTTAGATTCGAAGGTAGACCTGCCG**[TACCATCTCATGTGGAAGTATTATGAAA]**CACGAGACAGCGAAAAAATAGCCGACGTGGCCTATTACTGCATTATAG…TGGTATTGCTCATGTAAACACACCCAA[**TTTTAATACAAAAGAATTATTCATCCGCGG**]A[**ATAGATATCATTAAGCAGGGTCAAACAAAACTCACCAAAACGATAGG**]AACGCGAATTATGGAAGAATCCATGAAACTACGCCGCCCTGAGGACCATCGC…GCGCTGCAGCATCGCAATTTGCTGAGCCC[**GAGCCGGGAGAACGCTTCTCCTACGTTATCGTGGAAAAA**]CAGGTACAGTTTGATATCCAGGGCCACCGCACAGATTCCTCCAGAAAGGGGGACAAG[**ATGGAATACGTCTCTGAAGCAAAGGCTAAAAA**]TCTTCCTATTGATATA…

pp220

…TATTGTATAGTGCCGGGCGCCACTTGGGGGTAGGCTGGGTTGAAGGACTAAT[**AAACCTATCGGAGGGAAGTAATT**]GTGAGGATTGTGTATAGCCATCCTCATCAGGAAGAAT…CATGTCCCCAATCCGGGCAAA[**GGCATTGATGATATTTTTAAGCGCCTGAAAGTT]**AGAAAGAGAGCGCCCGATAAGGTCGC…TGCATGAGGCGTCCCGCAATAGCATCTCCGAGAATAG[**TGGCATAGTTTTCCTCGTAGGATTGAAACTCCTGTTTGTTATGCGTTAAATTGGAGTAAATCTGGGCCACATA]**ATAGTAATACATAAAGGTGTTAATTGCCT…CGAG[**AGCAGGAGTCATAAAGATTTTTAAAATTAGGGTCG]**GTTTTAGATATCTCCTCCAAAACATTTTT…ACAAGTTTG[**ATGCCTATTTTTTTCACATTTTCCAAAAAGTC]**GTTATAGGCTTGTGTGCTTTTATTCAAAAATTCCATGAGGA…AG[**TTTATCAAGTTCCTTTTGGGTGG]**GAGTTAAAATATTGTCAATAAAATTCGTTAAAATGTTGATTTGCAG[**GTTTTGTTCATTTAAAAGTCGACGATATACTGCTTCAATCATGGTGACTGC]**ATTAATGACTTCCTCATTGGGGGCTGC[**TTTGGTTACCTCCGTCACCATGCGCTC**]G

TGAAGTTGCTTAATGGCGTCGTTTAACAGCTTGATA[**TTTTCAAGTGTATTTTCTATACTGCC]**GTG…GCGGCCCCTTCGGTGGTATCTATA[**AGCTTATCCTGACCTAAATCAATAAATTCCTGGTTAATGGCGTCTGCAATCATTTTACAG]**ACGGTCTCCTGTTTTTCCGCATTTTTTACAAAGGTGGAAC[**CGGCTCGAGGATCGGGCAGTTGTTTTTTGATATCTTTAAGAATATCTTCGATGGGCTGCTTTGTGT]**CTA…GCCCTGATAATTCCTTCTATAATCCGCAG[**CTTTGCTTTACTCGATACGGAGTCTAT]**GTGATAATCTTT…

crRNA sequences (rU, rC, rA and rG indicate RNA nucleotides)

DNA Pol crRNA1

rUrArArUrUrUrCrUrArCrUrArArGrUrGrUrArGrArUrGrUrGrArGrUrUrUrUrGrUrUrUrGrArCrCrCrUrGrCrUrU

DNA Pol crRNA2

rUrArArUrUrUrCrUrArCrUrArArGrUrGrUrArGrArUrGrCrCrUrUrUrGrCrUrUrCrArGrArGrArCrGrUrArUrUrC

DNA Pol crRNA3

rUrArArUrUrUrCrUrArCrUrArArGrUrGrUrArGrArUrCrArCrGrArUrArArCrGrUrArGrGrArGrArArGrCrGrUrU

pp220 crRNA4

rUrArArUrUrUrCrUrArCrUrArArGrUrGrUrArGrArUrCrUrCrGrUrArGrGrArUrUrGrArArArCrUrCrCrUrGrUrU

pp220 crRNA5

rUrArArUrUrUrCrUrArCrUrArArGrUrGrUrArGrArUrArArArGrUrCrGrArCrGrArUrArUrArCrUrGrCrUrUrCrA

pp220 crRNA6

rUrArArUrUrUrCrUrArCrUrArArGrUrGrUrArGrArUrGrUrUrArCrCrUrCrCrGrUrCrArCrCrArUrGrCrGrCrUrC
