# Supplementary material for: Rapid detection of African swine fever virus using Cas12a-based portable paper diagnostics
Source: Cell Discov. 2020 Apr 7;6:18. doi: 10.1038/s41421-020-0151-5 (PMC7136273; doi:10.1038/s41421-020-0151-5)
Supplement: Supplementary file 4 — Supplementary Table S3 [file 41421_2020_151_MOESM4_ESM.pdf]

Supplementary Table S3 DNA oligos and primer sequences used

Oligonucleotides used to form dsDNA templates for *in vitro transcription*

Oligo	Sequence (5' to 3')
DNA polymerase crRNA1-F	GAAATTAATACGACTCACTATAGGTAATTTCTACTAAG TGTAGATGTGAGTTTTGTTTGACCCTGCTT
DNA polymerase crRNA1-R	AAGCAGGGTCAAACAAAACTCACATCTACACTTAGTA GAAATTACCTATAGTGAGTCGTATTAATTTC
DNA polymerase crRNA2-F	GAAATTAATACGACTCACTATAGGTAATTTCTACTAAG TGTAGATGCCTTTGCTTCAGAGACGTATTC
DNA polymerase crRNA2-R	GAATACGTCTCTGAAGCAAAGGCATCTACACTTAGTA GAAATTACCTATAGTGAGTCGTATTAATTTC
DNA polymerase crRNA3-F	GAAATTAATACGACTCACTATAGGTAATTTCTACTAAG TGTAGATCACGATAACGTAGGAGAAGCGTT
DNA polymerase crRNA3-R	AACGCTTCTCCTACGTTATCGTGATCTACACTTAGTA GAAATTACCTATAGTGAGTCGTATTAATTTC
PP220 crRNA1-F	GAAATTAATACGACTCACTATAGGTAATTTCTACTAAG TGTAGATCTCGTAGGATTGAAACTCCTGTT
PP220 crRNA1-R	AACAGGAGTTTCAATCCTACGAGATCTACACTTAGTA GAAATTACCTATAGTGAGTCGTATTAATTTC
PP220 crRNA2-F	GAAATTAATACGACTCACTATAGGTAATTTCTACTAAG TGTAGATAAAGTCGACGATATACTGCTTCA
PP220 crRNA2-R	TGAAGCAGTATATCGTCGACTTTATCTACACTTAGTA GAAATTACCTATAGTGAGTCGTATTAATTTC

PP220 crRNA3-F	GAAATTAATACGACTCACTATAGGTAATTTCTACTAAGTGTAGATGTTACCTCCGTCACCATGCGCTC
PP220 crRNA3-R	GAGCGCATGGTGACGGAGGTAACATCTACACTTAGTAGAAATTACCTATAGTGAGTCGTATTAATTTC

WT, PAM-mutated or spacer targeting region-mutated DNA oligos (targeted by crRNA1)

WT-NTS

TTCGCGTTCCTATCGTTTTGGTGAGTTTTGTTTGACCCTGCTTAATGATATCTATTCCG

WT-TS

CGGAATAGATATCATTAAGCAGGGTCAAACAAAACTCACCAAAACGATAGGAACGCGAA

PAM mut NTS

TTCGCGTTCCTATCGTAGCGGTGAGTTTTGTTTGACCCTGCTTAATGATATCTATTCCG

PAM mut TS

CGGAATAGATATCATTAAGCAGGGTCAAACAAAACTCACCGCTACGATAGGAACGCGAA

1-2 mut NTS

TTCGCGTTCCTATCGTTTTGCAGAGTTTTGTTTGACCCTGCTTAATGATATCTATTCCG

1-2 mut TS

CGGAATAGATATCATTAAGCAGGGTCAAACAAAACTCTGCAAAACGATAGGAACGCGAA

3-4 mut NTS

TTCGCGTTCCTATCGTTTTGGTCTGTTTTGTTTGACCCTGCTTAATGATATCTATTCCG

3-4 mut TS

CGGAATAGATATCATTAAGCAGGGTCAAACAAAACAGACCAAAACGATAGGAACGC
GAA
5-6 mut NTS
TTCGCGTTCCTATCGTTTTGGTGACATTTGTTTGACCCTGCTTAATGATATCTATTCC
G
5-6 mut TS
CGGAATAGATATCATTAAGCAGGGTCAAACAAATGTCACCAAAACGATAGGAACGCG
AA
7-8 mut NTS
TTCGCGTTCCTATCGTTTTGGTGAGTAATGTTTGACCCTGCTTAATGATATCTATTCC
G
7-8 mut TS
CGGAATAGATATCATTAAGCAGGGTCAAACATTACTCACCAAAACGATAGGAACGCG
AA
9-10 mut NTS
TTCGCGTTCCTATCGTTTTGGTGAGTTTACTTTGACCCTGCTTAATGATATCTATTCC
G
9-10 mut TS
CGGAATAGATATCATTAAGCAGGGTCAAAGTAAACTCACCAAAACGATAGGAACGCG
AA
11-12 mut NTS
TTCGCGTTCCTATCGTTTTGGTGAGTTTTGAATGACCCTGCTTAATGATATCTATTCC
G
11-12 mut TS
CGGAATAGATATCATTAAGCAGGGTCATTCAAAACTCACCAAAACGATAGGAACGCG
AA
13-14 mut NTS
TTCGCGTTCCTATCGTTTTGGTGAGTTTTGTTACACCCTGCTTAATGATATCTATTCC
G
13-14 mut TS
CGGAATAGATATCATTAAGCAGGGTGTAACAAAACTCACCAAAACGATAGGAACGCG

AA

15-16 mut NTS

TTCGCGTTCCTATCGTTTTGGTGAGTTTTGTTTGTGCCTGCTTAATGATATCTATTCC
G

15-16 mut TS

CGGAATAGATATCATTAAGCAGGCACAAACAAAACTCACCAAAACGATAGGAACGCG
AA

17-18mut NTS

TTCGCGTTCCTATCGTTTTGGTGAGTTTTGTTTGACGGTGCTTAATGATATCTATTCC
G

17-18 mut TS

CGGAATAGATATCATTAAGCACCGTCAAACAAAACTCACCAAAACGATAGGAACGCG
AA

19-20 mut NTS

TTCGCGTTCCTATCGTTTTGGTGAGTTTTGTTTGACCCACCTTAATGATATCTATTCC
G

19-20 mut TS

CGGAATAGATATCATTAAGGTGGGTCAAACAAAACTCACCAAAACGATAGGAACGCG
AA

WT, PAM-mutated or spacer targeting region-mutated DNA oligos (targeted by crRNA5)

WT-NTS

TTGCAGGTTTTGTTCATTTAAAAGTCGACGATATACTGCTTCAATCATGGTGACTGCA
T

WT-TS

ATGCAGTCACCATGATTGAAGCAGTATATCGTCGACTTTTAAATGAACAAAACCTGCA
A

PAM mut NTS

TTGCAGGTTTTGTTCAAGCAAAAGTCGACGATATACTGCTTCAATCATGGTGACTGC
AT

PAM mut TS

ATGCAGTCACCATGATTGAAGCAGTATATCGTCGACTTTTGCTTGAACAAAACCTGC
AA

1-2 mut NTS

TTGCAGGTTTTGTTCATTTATTAGTCGACGATATACTGCTTCAATCATGGTGACTGCA
T

1-2 mut TS

ATGCAGTCACCATGATTGAAGCAGTATATCGTCGACTAATAAATGAACAAAACCTGCA
A

3-4 mut NTS

TTGCAGGTTTTGTTCATTTAAATCTCGACGATATACTGCTTCAATCATGGTGACTGCA
T

3-4 mut TS

ATGCAGTCACCATGATTGAAGCAGTATATCGTCGAGATTTAAATGAACAAAACCTGCA
A

5-6 mut NTS

TTGCAGGTTTTGTTCATTTAAAAGAGGACGATATACTGCTTCAATCATGGTGACTGCA
T

5-6 mut TS

ATGCAGTCACCATGATTGAAGCAGTATATCGTCCTCTTTTAAATGAACAAAACCTGCA
A

7-8 mut NTS

TTGCAGGTTTTGTTCATTTAAAAGTCCTCGATATACTGCTTCAATCATGGTGACTGCA
T

7-8 mut TS

ATGCAGTCACCATGATTGAAGCAGTATATCGAGGACTTTTAAATGAACAAAACCTGC
AA

9-10 mut NTS

TTGCAGGTTTTGTTCATTTAAAAGTCGAGCATATACTGCTTCAATCATGGTGACTGCA
T

9-10 mut TS

ATGCAGTCACCATGATTGAAGCAGTATATGCTCGACTTTTAAATGAACAAAACCTGCA

A

11-12 mut NTS

TTGCAGGTTTTGTTCATTTAAAAGTCGACGTAATACTGCTTCAATCATGGTGACTGCA
T

11-12 mut TS

ATGCAGTCACCATGATTGAAGCAGTATTACGTCGACTTTTAAATGAACAAAACCTGC
AA

13-14 mut NTS

TTGCAGGTTTTGTTCATTTAAAAGTCGACGATTAACTGCTTCAATCATGGTGACTGCA
T

13-14 mut TS

ATGCAGTCACCATGATTGAAGCAGTTAATCGTCGACTTTTAAATGAACAAAACCTGC
AA

15-16 mut NTS

TTGCAGGTTTTGTTCATTTAAAAGTCGACGATATTGTGCTTCAATCATGGTGACTGCA
T

15-16 mut TS

ATGCAGTCACCATGATTGAAGCACAATATCGTCGACTTTTAAATGAACAAAACCTGC
AA

17-18 mut NTS

TTGCAGGTTTTGTTCATTTAAAAGTCGACGATATACACCTTCAATCATGGTGACTGCA
T

17-18 mut TS

ATGCAGTCACCATGATTGAAGGTGTATATCGTCGACTTTTAAATGAACAAAACCTGCA
A

19-20 mut NTS

TTGCAGGTTTTGTTCATTTAAAAGTCGACGATATACTGGATCAATCATGGTGACTGCA
T

19-20 mut TS

ATGCAGTCACCATGATTGATCCAGTATATCGTCGACTTTTAAATGAACAAAACCTGCA
A

Primers used in PCR, RPA and qPCR

DNA Pol PCR primers

F: TGCGCCGAGGTGAATGAATA

R: TGTCCCCCTTTCTGGAGGAA

pp220 PCR primers

F TTCTGCGGAGACAAGACCAC

R GCCGCCAGTATATGTCGACA

RPA primers

F TATTGCTCATGTAAACACACCCAATTTTAATAC

R GTAGTTTCATGGATTCTTCCATAATTCGCGTT

ASFV qPCR primers and probe

P72-qPCR probe FAM-CCACGGGAGGAATACCAACCCAGTG-TAMRA

P72 primer-F CTGCTCATGGTATCAATCTTATCGA

P72 primer-R GATACCACAAGATCAGCCGT
